# eIF4E Overexpression Is Associated with Poor Prognoses of Ovarian Cancer

**DOI:** 10.1155/2020/8984526

**Published:** 2020-12-12

**Authors:** Jun Zheng, Xueqing Li, Chunyan Zhang, Yiqiang Zhang

**Affiliations:** ^1^Department of Biochemistry, Changzhi Medical College, Changzhi, Shanxi, China; ^2^Department of Gynaecology and Obstetrics, Heping Hospital Affiliated to Changzhi Medical College, Changzhi, Shanxi, China

## Abstract

**Aim:**

Ovarian cancer is a common malignant tumor of the gynecological oncology worldwide, with a high incidence and mortality rate and poor prognosis. Searching for new diagnostic molecular biomarkers for ovarian cancer is extremely significant.

**Methods:**

Here, we analyzed the expression rates of eIF4E and cyclin D1 proteins in 123 cases of cancer tissue samples and 38 cases of paracancerous tissue samples and studied the connection between the expression rates of eIF4E and cyclin D1 proteins by immunohistochemistry and statistically correlated with clinicopathological features in ovarian cancer.

**Results:**

The results showed that the expression rates of eIF4E and cyclin D1 proteins in ovarian cancer tissues were significantly higher than those in noncancerous epithelial ovarian tissues (*P* = 0.001 and *P* = 0.032, respectively). Additionally, the results revealed that a higher expression rate of eIF4E (*P* = 0.008) was found in the advanced stage (stage III/IV), and also patients with cervical lymph node metastasis displayed higher expression of eIF4E (*P* < 0.001) and cyclin D1 (*P* = 0.033) than those without lymph node metastasis. Spearman's rank correlation test showed that there was a significant positive correlation between the eIF4E and cyclin D1 proteins in ovarian cancer. The Kaplan-Meier method showed that patients with lower expression of eIF4E had marginally better survival than those with high expression of eIF4E (*P* = 0.012). Multivariate Cox regression analysis further identified that positive expression of eIF4E was an independent prognostic factor.

**Conclusion:**

In ovarian cancer, eIF4E might be a valuable biomarker to predict poor prognoses and a potential therapeutic target to develop valid treatment strategies.

## 1. Introduction

Ovarian cancer is a common type of gynecological cancer worldwide, with a high incidence and mortality rate. Pieces of evidence indicate that ovarian cancer remains the fifth cause of cancer-related death among women in the United States. Over the last three decades, the five-year survival rate of ovarian cancer patients has remained at a low level with 35-46% [1, 2]. The high mortality rate and poor prognosis of ovarian cancer patients are due to the lack of early symptoms and early effective screening tests. So, most patients are first diagnosed at the advanced stage or metastasis. In addition, although traditional therapy such as platinum-based treatment has been discovered more than 30 years ago, the overall survival rate of women has changed little now because of the advanced resistance. Although these patients have been treated with first-line surgery and chemotherapy, most of them will still face relapse and death [3, 4]. So far, there are no reliable molecular markers to predict aggressive phenotypes. Therefore, searching for new molecular biomarkers for ovarian cancer is extremely urgent, which may provide a potential screening test for early detection and reveal a novel therapeutic strategy to improve the survival of ovarian cancer patients.

**Figure 1:**
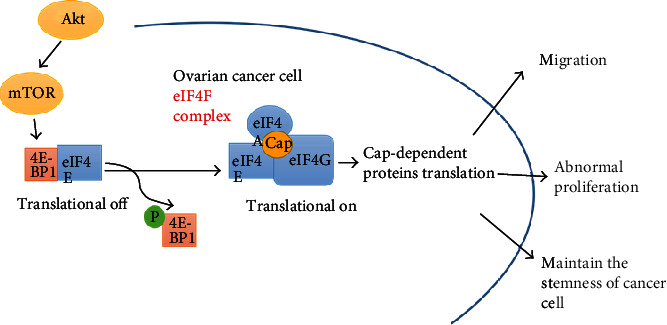
When the 4EBP1 protein is phosphorylated by the AKT/mTOR signal pathway, eIF4E will be free from 4EBP1 to bind with eIF4G and then form the eIF4F complex, which subsequently promotes the translation of cap-dependent proteins. These special proteins could promote migration and abnormal proliferation and maintain the stemness of ovarian cancer cells.

Protein translational regulation is an important strategy to control gene expression in normal eukaryotic cells. Abnormal expression of specific proteins such as c-MYC and cyclin D1 can lead to tumorigenesis [5, 6]. The main target of the translational regulation factor is eukaryotic translation initiation factor 4E (eIF4E), which has been considered a limiting factor in the regulation of gene expression and affects many essential cellular processes in eukaryotic cells because it is the least abundant initiator factor involved in the eIF4F complex [7, 8]. The activity of eIF4E is regulated by multiple aspects. The 4EBP1 profilin of eIF4E prevents the assembly of eIF4F by competing with eIF4G to bind to eIF4E. When the 4EBP1 protein is phosphorylated by the AKT/mTOR signal pathway, eIF4E will be free from 4EBP1 to bind with eIF4G and then form the 4F complex, which subsequently promotes the translation of specific proteins [9] (shown in Figure 1). Overexpression of eIF4E is the main mechanism for eIF4E activation and has been discovered in many cancer tissues. Under physiological conditions, eIF4E with a low expression level can play a normal role in cells. When eIF4E is overexpressed, it can selectively affect the translation rate of some certain proteins, thus inducing cell proliferation and promoting the antiapoptosis, invasion, recurrence, and metastasis of tumor cells. Previous studies have confirmed that eIF4E was overexpressed in multiple tumor tissues including breast, lung, head, and neck tumors [9–13]. Hence, overexpression of eIF4E might be a biological marker for malignant tumors, with clinical significance of poor prognoses. We believed that changes in the eIF4E level might affect the translation rate of certain proteins, particularly those related to cell growth and survival involved in oncogenesis, invasion, and metastasis. Some studies have shown that eIF4E was correlated with the occurrence and development of human cancers, and the inhibition of eIF4E expression acted as a potential therapeutic target [13–18]. Previous studies have substantiated that the expression of eIF4E protein was closely related to clinicopathologic features and poor prognoses [19]. However, whether overexpression of eIF4E is associated with the development and progression of ovarian cancer has not been reported. In the current study, we analyzed the expression of eIF4E and cyclin D1 proteins in 123 ovarian cancer specimens and 38 noncancerous epithelial ovarian specimens by immunohistochemistry (IHC) and investigated the correlation between the expression of these two proteins and clinicopathologic/prognostic characteristics in ovarian cancer.

## 2. Materials and Methods

### 2.1. Tissue Samples and Clinical Data

One hundred and twenty-three (123) cases of paraffin-embedded ovarian cancer tissues and thirty-eight (38) cases of paracancerous tissues were collected from the Peace Hospital Affiliated to Changzhi Medical College (Changzhi, China). The patients' age was between 35 and 73 years (median, 51.8 years). All specimens and protocols were approved by the Ethics Review Board with informed consent. All patients had not received chemotherapy/radiotherapy before the biopsy. All samples were fixed with 4% neutral formalin and embedded with paraffin. Clinical data including age, clinical stages, lymph node metastasis, and survival status were available for each patient as shown in Table 1.

### 2.2. IHC and Scores

The IHC staining for eIF4E and cyclin D1 proteins in ovarian cancer samples was carried out with ready-to-use EnVision™+ Dual Link System-HRP methods (Dako, USA). Briefly, each section was regularly dewaxed through xylene followed by rehydration with a gradient of alcohols; subsequently, high-temperature antigen retrieval was achieved by heating the sections for 15 min in a 10 mM citrate buffer (pH 6.0) in a microwave, and then the sections were immersed into 3% hydrogen peroxide (H_2_O_2_) in methanol for 15 min. Sections were incubated with 5% normal goat serum for 30 min at room temperature. After that, the sections were incubated with a primary antibody, including eIF4E (pS473) protein (Epitomics, Inc., USA) in 1 : 200 dilution and cyclin D1 protein (Cell Signaling, USA) in 1 : 200 dilution at 4°C overnight, and washed thoroughly with PBS, and a secondary antibody (Maixin Biotechnology Inc., China) conjugated with a labeled polymer-HRP was incubated at room temperature for 50 minutes. The color reaction was developed by using DAB (Maixin Biotechnology Inc., China). Positive and negative controls were included in every experiment.

Immunohistochemical staining was estimated independently by two pathologists. The eIF4E and cyclin D1 staining was scored as negative (<10% staining) and positive (>10% staining), respectively [19].

### 2.3. Statistical Analyses

The chi-squared test was used to evaluate the relationship between the expression of eIF4E and cyclin D1 and clinicopathological characteristics. The Kaplan-Meier method was used to calculate the survival curves. Spearman's rank correlation coefficient was performed to assess the relationship between the eIF4E and cyclin D1 proteins. The Cox proportional hazard regression model was used to evaluate whether the expression rates of eIF4E and cyclin D1 proteins were independent prognostic factors. For all analyses, *P* values were two-sided and *P* < 0.05 was considered statistically significant. All statistical analyses were performed using SPSS 18.0 (Statistical Package for the Social Sciences, Version 18.0).

## 3. Results

### 3.1. eIF4E and Cyclin D1 Proteins Were Overexpressed in Ovarian Cancer Tissues

**Figure 2:**
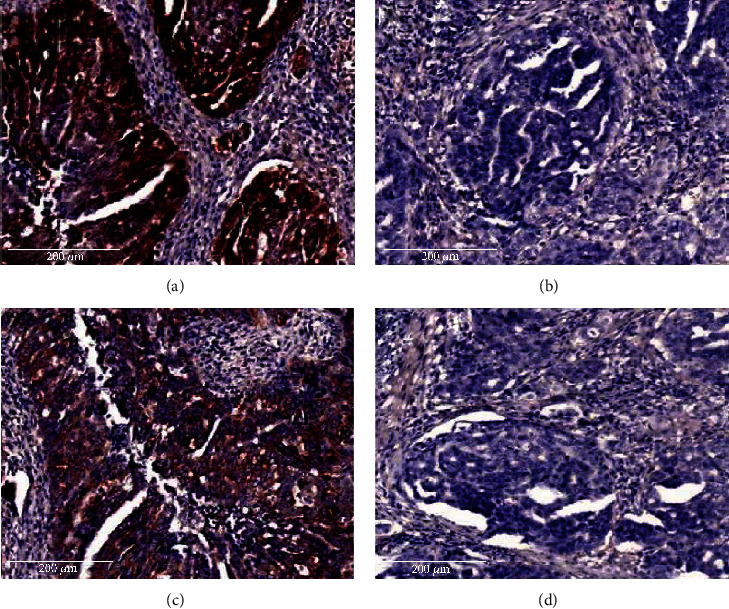
Overexpression of eIF4E and cyclin D1 proteins in ovarian cancer tissues. The expression of eIF4E and cyclin D1 proteins was detected by immunohistochemistry using specific antibodies as described in Materials and Methods. Strong positive ((a), DAB staining) and negative ((b), DAB staining) expression rates of eIF4E protein were shown in the ovarian cancer tissues. Strong positive ((c), DAB staining) and negative ((d), DAB staining) expression rates of cyclin D1 protein were shown in ovarian cancer tissues.

We examined the cellular location and expression level of eIF4E and cyclin D1 proteins in ovarian cancer tissues by IHC. Strong positive staining of eIF4E was found in the cytoplasm and nucleus of ovarian cancer tissues (Figure 2). And positive staining of cyclin D1 was identified in the cell cytoplasm of ovarian cancer tissues. As shown in Table 2, the positive expression rate of eIF4E and cyclin D1 in ovarian cancer tissues was 68.3% (84/123) and 64.2% (79/123), respectively. And in noncancerous epithelial ovarian tissues, the expression rate was 39.5% (15/38) and 44.7% (17/38), respectively. The expression rate of eIF4E and cyclin D1 in ovarian cancer tissues was significantly higher than that in noncancerous epithelial ovarian tissues (*P* = 0.001 and *P* = 0.032, respectively).

### 3.2. eIF4E and Cyclin D1 Proteins Were Related to Clinicopathological Features of Ovarian Cancer

We further studied the correlation between the expression of eIF4E and cyclin D1 proteins and clinicopathological features of ovarian cancer, including age, clinical stage, lymph node metastasis status, and survival status by a univariate chi-squared test (Table 1). Statistical analyses revealed that higher positive expression of eIF4E (*P* = 0.008) was found in the advanced (III/IV) stage than the early (I/II) stage, and patients with cervical lymph node metastasis had higher expression of eIF4E (*P* < 0.001) and cyclin D1 (*P* = 0.033) than those without lymph node metastasis. The results also showed that strong positive expression of eIF4E was related to the survival status of ovarian cancer patients (*P* = 0.001). However, the expression of cyclin D1 had no significant differences with the clinical stage (I/II or III/IV) and survival status (*P* > 0.05, respectively). Moreover, the positive expression of eIF4E and cyclin D1 proteins was not related to the age of ovarian cancer patients (*P* > 0.05, respectively).

### 3.3. eIF4E Had a Positive Correlation with Cyclin D1 Proteins in Ovarian Cancer

We further analyzed the correlation between the expression rates of eIF4E and cyclin D1 proteins in ovarian cancer using Spearman's rank correlation test (Table 3). Data showed that there was a strong positive correlation between the eIF4E and cyclin D1 proteins (*r* = 2.453, *P* = 0.002).

### 3.4. Overexpression of eIF4E Protein Was Associated with Poor Prognoses

**Figure 3:**
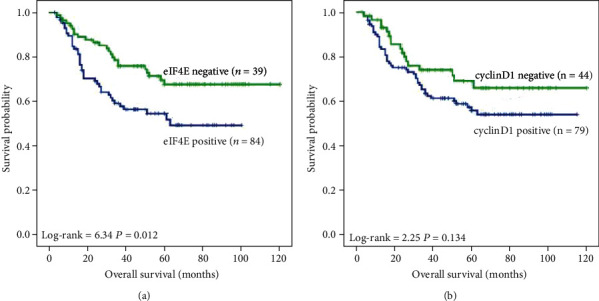
Kaplan-Meier overall survival curves of ovarian cancer patients. Kaplan-Meier analysis was used to plot the survival curve of all 123 ovarian cancer patients with expression of eIF4E and cyclin D1 proteins, and statistical significance was assessed using the log-rank test. (a) Kaplan-Meier curves showed that worse overall survival was seen in eIF4E-positive patients compared with eIF4E-negative patients (*P* = 0.012, two-sided). (b) Kaplan-Meier curves showed that the expression of cyclin D1 protein had no significant correlation with overall survival rates of ovarian cancer patients (*P* > 0.05, two-sided).

We obtained the follow-up data of all patients and studied the association between the expression of eIF4E and cyclin D1 proteins and overall survival using the Kaplan-Meier method.  Figure 3 displays some evidence of survival advantage for ovarian cancer patients with negative expression of eIF4E and cyclin D1. The ovarian cancer patients with lower expression of eIF4E had a better survival rate than those with strong expression of eIF4E (*P* = 0.012, Figure 3(a)). Although patients had better survival outcomes with negative expression of cyclin D1, it was not statistically significant (*P* > 0.05, Figure 3(b)). Meanwhile, we investigated potential correlations of independent prognostic factors including clinical stages, lymph node metastasis status, and expression of eIF4E and cyclin D1 proteins for ovarian cancer patients by multivariate Cox proportional hazard regression analysis.  Table 4 reveals that positive expression of eIF4E protein was identified as an independent poor prognostic factor for ovarian cancer patients (*P* = 0.003), as well as clinical stages (*P* = 0.002) and lymph node metastasis status (*P* = 0.001). Nevertheless, cyclin D1 protein was not an independent poor prognostic factor (*P* > 0.05).

## 4. Discussion and Conclusion

Increasing pieces of evidence have shown that eIF4E is a speed limiting factor in the regulation of gene expression and affects many essential cellular processes, including cell growth, proliferation, cell differentiation, and survival in eukaryotic cells [8, 13, 16]. Overexpression of eIF4E has been reported in many malignant carcinomas, such as breast carcinoma, prostate cancer, nasopharyngeal carcinoma, lung carcinoma, and head and neck carcinoma, and was associated with clinicopathological features and prognostic implications [11, 14, 20–24]. Several *in vitro* experiments showed that a high level of eIF4E was associated with cell proliferation and cancer progression and significantly reduced the level of antiapoptotic proteins which induced apoptosis and sensitized the cells to chemotherapy [25]. Previous studies have demonstrated that the knockdown of eIF4E led to the prolongation of G1 phase transition and G0/G1 cell cycle arrest by decreasing the translation of c-MYC and cyclin D1 in some carcinoma cell lines [26, 27]. A similar effect was observed in *in vivo* experiment where antisense oligonucleotide (ASO) selectively reduced the eIF4E level in human tumor xenografts and dramatically decreased tumor growth without eliciting cytotoxicity in normal tissues [28, 29]. In the previous study, the results showed that there was high positive expression of eIF4E in nasopharyngeal carcinoma tissues and it was closely related to clinicopathologic features and poor prognoses [19]. In this study, the results showed that there was higher positive expression of eIF4E and cyclin D1 in ovarian cancer than in noncancerous epithelial ovarian tissues, and eIF4E had a strong positive correlation with cyclin D1 protein. Cyclin D1 is putatively one of the weak mRNA oncogenes, and its expression is effectively increased by eIF4E overexpression [30, 31]. The result of eIF4E overexpression in ovarian cancer was consistent with other reports. This finding strongly indicated that eIF4E might play a crucial role in the carcinogenesis of ovarian cancer.

Tumor progression is related to recurrence and metastasis and also affects patients' prognoses. Recently, a study found that the eIF4E could enhance MMP9 expression to promote metastasis in mouse breast cancer [32]. Other studies indicated that the treatment with 4EGI1 (a competitive eIF4E/eIF4G interaction inhibitor) might affect the expression of both eIF4E and cyclin D1 in the cellular level of NPC [32]. However, whether eIF4E could promote metastasis in ovarian cancer tissues is still unknown. In our study, results showed that higher expression of eIF4E protein was found in patients with the advanced (III/IV) stage and cervical lymph node metastasis. The expression of eIF4E was negatively correlated with the survival status of patients. These results indicated that the expression of eIF4E is important in the progression of ovarian cancer. We found that the expression of cyclin D1 was higher in patients with cervical lymph node metastasis than those without lymph node metastasis, while it had no significant difference in the clinical stage (I/II or III/IV) and survival.

There are many factors related to prognoses such as lymph node metastasis and clinical stage. eIF4E protein overexpression has been observed in many solid tumors and was correlated with recurrence, metastasis, and poor prognoses of human tumors [11, 14, 20–24]. Our results showed that patients with lower expression of eIF4E had better survival than those with strong expression. Meanwhile, multivariate analysis proved that eIF4E positive expression was an independent factor of ovarian cancer. Therefore, eIF4E may act as a novel prognostic molecular marker and be a potential molecular target for the therapy and prevention of ovarian cancer.

In summary, our results proved that the expression of eIF4E protein was higher in ovarian cancer tissues and might promote the proliferation and cell cycle progression by enhancing the translation of cyclin D1. Overexpression of eIF4E was associated with clinicopathological features and prognoses of ovarian cancer patients and might be a novel valuable biomarker to predict poor prognoses and a therapeutic target to develop valid treatment strategies. *In vitro* studies can improve the understanding of the molecular mechanisms involved. It is of great value and urgency to further study the relevant aspects. In ovarian cancer, eIF4E might be a valuable biomarker to predict poor prognoses and may be a therapeutic target to develop valid treatment strategies.

## Figures and Tables

**Table 1:** Association between the expression of eIF4E and cyclin D1 proteins and clinicopathological features of ovarian cancer patients.

Characteristics	eIF4E			Cyclin D1			P-4EBP1		
	+ (%)	- (%)	*P*	+ (%)	- (%)	*P*	+ (%)	- (%)	*P*
Age (yr)									
≤55 (*n* = 48)	32 (66.7)	16 (33.3)	0.096	30 (62.5)	18 (37.5)	0.102	26 (54.2)	22 (45.8)	0.008
>55 (*n* = 75)	52 (69.3)	23 (30.7)	0.757	49 (65.3)	26 (34.7)	0.749	40 (53.3)	35 (46.7)	0.928
Clinical stages									
I-II (*n* = 37)	19 (51.4)	18 (48.6)	7.014	25 (67.6)	12 (32.4)	0.257	17 (45.9)	20 (50.1)	1.266
III-IV (*n* = 86)	65 (75.6)	21 (24.4)	0.008	54 (62.8)	32 (37.2)	0.612	49 (57.0)	37 (43.0)	0.261
LN status									
LNM (*n* = 82)	68 (82.9)	14 (17.1)	24.330	58 (70.7)	24 (29.3)	4.519	53 (64.6)	29 (35.4)	11.917
No LNM (*n* = 41)	16 (39.0)	25 (61.0)	<0.001	21 (51.2)	20 (48.8)	0.033	13 (31.7)	28 (68.3)	0.001
Survival status									
Dead (*n* = 88)	68 (77.3)	20 (22.7)	11.517	59 (67.0)	29 (33.0)	1.069	55 (62.5)	33 (37.5)	
Alive (*n* = 35)	16 (45.7)	19 (54.3)	0.001	20 (57.1)	15 (42.9)	0.301	11 (31.4)	24 (68.6)	0.002

Abbreviations: LN: lymph node; LNM: lymph node metastasis. A chi-squared test was used to evaluate the relationship between the expression of eIF4E and cyclin D1 and clinicopathological characteristics.

**Table 2:** The expression of eIF4E and cyclin D1 proteins in ovarian cancer tissues and noncancerous ovarian tissues.

Histologic type	eIF4E		Cyclin D1	
	+ (%)	- (%)	+ (%)	- (%)
OC (*n* = 123)	84 (68.3)	39 (31.7)	79 (64.2)	44 (35.8)
NOC (38)	15 (39.5)	23 (60.5)	17 (44.7)	21 (55.3)
*χ* ^2^	10.182		4.581	
*P*	0.001		0.032	

Abbreviations: OC: ovarian cancer tissues; NOC: noncancerous ovarian tissues. A chi-squared test was used to detect the difference of expression of eIF4E and cyclin D1 proteins in ovarian cancer tissues and noncancerous ovarian tissues.

**Table 3:** The pairwise association between the expression of eIF4E and cyclin D1 proteins in ovarian cancer patients.

eIF4E	Cyclin D1		
	Positive (%)	Negative (%)	*P* value
Positive (%)	66 (53.7)	18 (14.6)	0.002
Negative (%)	13 (10.6)	26 (21.1)	(*r* = 2.453)

Spearman's rank correlation coefficient was performed to assess the relationship between the eIF4E and cyclin D1 proteins.

**Table 4:** Summary of multivariate analysis of the Cox proportional hazard model for overall survival of ovarian cancer patients.

	SE	Wald	*P* value	Exp(*B*)	95% CI
eIF4E expression	0.274	8.785	0.003^∗^	0.444	0.260-0.760
Cyclin D1 expression	0.507	0.130	0.719	1.200	0.445-3.240
LN status (LNM vs. no LNM)	0.384	10.561	0.001^∗^	3.480	1.640-7.383
Clinical stages (I-II vs. III-IV)	0.325	9.201	0.002^∗^	4.403	2.194-8.836

Abbreviations: LN: lymph node; LNM: lymph node metastasis. The Cox proportional hazard regression model was used to evaluate whether the expression rates of eIF4E and cyclin D1 proteins were independent prognostic factors.

## Data Availability

All data generated or analyzed during this study are included in this published article.
